# Population Estimation Using a 3D City Model: A Multi-Scale Country-Wide Study in the Netherlands

**DOI:** 10.1371/journal.pone.0156808

**Published:** 2016-06-02

**Authors:** Filip Biljecki, Ken Arroyo Ohori, Hugo Ledoux, Ravi Peters, Jantien Stoter

**Affiliations:** 3D Geoinformation, Delft University of Technology, Delft, The Netherlands; Boston University, UNITED STATES

## Abstract

The remote estimation of a region’s population has for decades been a key application of geographic information science in demography. Most studies have used 2D data (maps, satellite imagery) to estimate population avoiding field surveys and questionnaires. As the availability of semantic 3D city models is constantly increasing, we investigate to what extent they can be used for the same purpose. Based on the assumption that housing space is a proxy for the number of its residents, we use two methods to estimate the population with 3D city models in two directions: (1) disaggregation (areal interpolation) to estimate the population of small administrative entities (e.g. neighbourhoods) from that of larger ones (e.g. municipalities); and (2) a statistical modelling approach to estimate the population of large entities from a sample composed of their smaller ones (e.g. one acquired by a government register). Starting from a complete Dutch census dataset at the neighbourhood level and a 3D model of all 9.9 million buildings in the Netherlands, we compare the population estimates obtained by both methods with the actual population as reported in the census, and use it to evaluate the quality that can be achieved by estimations at different administrative levels. We also analyse how the volume-based estimation enabled by 3D city models fares in comparison to 2D methods using building footprints and floor areas, as well as how it is affected by different levels of semantic detail in a 3D city model. We conclude that 3D city models are useful for estimations of large areas (e.g. for a country), and that the 3D approach has clear advantages over the 2D approach.

## Introduction

Geographic information science (GIS) and demography have long been closely related, and GIS techniques are ubiquitous in mapping, analysing, and filling gaps in demographic data. In particular, geostatistical techniques are often used to estimate a region’s population in the absence of reliable or complete census data [1, 2].

*2D GIS datasets* (e.g. satellite imagery and maps) have been used extensively in the past 50 years for this purpose, as several of them have been found to be reasonable proxies for population [2–21]. For instance, Bakillah et al. [22] and Doll et al. [23] estimate the population based on the concentration of surrounding points of interest (e.g. restaurants); Anderson et al. [24] and Sutton [25] use night-time imagery following the hypothesis that city lights indicate the magnitude of the urban extent, which in turn indicates the population. Pozzi and Small [26] infer the population density from a vegetation cover map, based on the idea that less vegetation means more people; Xie [27] finds the relation between the density of road network and population; Steiger et al. [28] analyse georeferenced Twitter data to locate clusters indicating home- and work-related social activities that can serve as a proxy to estimate the residential and workplace population census data; and Lwin et al. [29] do a similar work using geolocated mobile phone usage data.

Among all these methods, many successful approaches rely on 2D datasets (maps) containing *building footprints* (e.g. derived from cadastral records or satellite imagery). The simplest approaches rely on the total number of buildings in a region or the total area of building footprints in it [30–32]. These methods perform reasonably well in homogeneous areas, but they exhibit significant errors in areas where buildings have a great variation in the number of storeys.

With the advancement of remote sensing technologies, such as lidar and aerial photogrammetry [33–37], it is now possible to automatically and remotely measure the height of a building, which can be used to obtain a volumetric representation of a building (3D city model) that is useful for population estimates. In fact, several researchers have indicated that the volume of buildings and the floorspace provide a strong cue for its population [5, 15, 22, 30, 38–46]. For example, Lu et al. [39] use multiple regression models to perform a study in Denver, Colorado, based on both footprint areas and building volumes. Lwin and Murayama [44] and Alahmadi et al. [46, 47] estimate the number of floors from an elevation dataset, and multiply it with the footprint area to get the approximate internal area of the apartments. Their results indicate that the volume-based approach gives more accurate results than the area of the footprints due to heterogeneous building morphologies.

However, despite the frequent indication that volume-based methods can improve on the estimates of area-based methods, there has been no large-scale study that conclusively proves that this is true. Existing studies have several gaps: they usually focus on single metropolitan areas, which can be relatively homogeneous; they seldom compare the accuracy of different approaches within the same region; they derive a building’s volume based on a raster dataset, which limits its accuracy; they do not consider how this approach scales between larger and smaller areas; and they do not consider how the level of detail of the used volumetric representation affects the accuracy of the result.

The goal of this paper is to bridge these gaps. We investigate *to what extent 3D city models can be used to estimate the population of a region* by performing a multi-scale country-wide study in the Netherlands. As the Dutch government provides both highly accurate census and building data, we consider that the Netherlands serves as an excellent case study, both for the experiments and the validation of the methods.

We therefore evaluate the use of 3D city models in population estimation in two directions: (1) disaggregation (areal interpolation) to estimate the population of small administrative entities (e.g. neighbourhoods) from that of larger ones (e.g. municipalities); and (2) a statistical modelling approach to estimate the population of large entities from a sample composed of their smaller ones (e.g. one acquired by a government register). We compare the population estimates obtained by both methods with the actual population as reported in the census, and use it to evaluate the quality that can be achieved by estimations at different administrative levels. We also analyse how the volume-based estimation enabled by 3D city models fares in comparison to 2D methods using building footprints and floor areas, as well as how it is affected by different levels of semantic detail (information on building use) in a 3D city model. We conclude that 3D city models are useful for large scale estimations (e.g. for a country), and that the 3D approach has clear advantages over the 2D approach.

## Materials and Methods

### Census data

**Fig 1:**
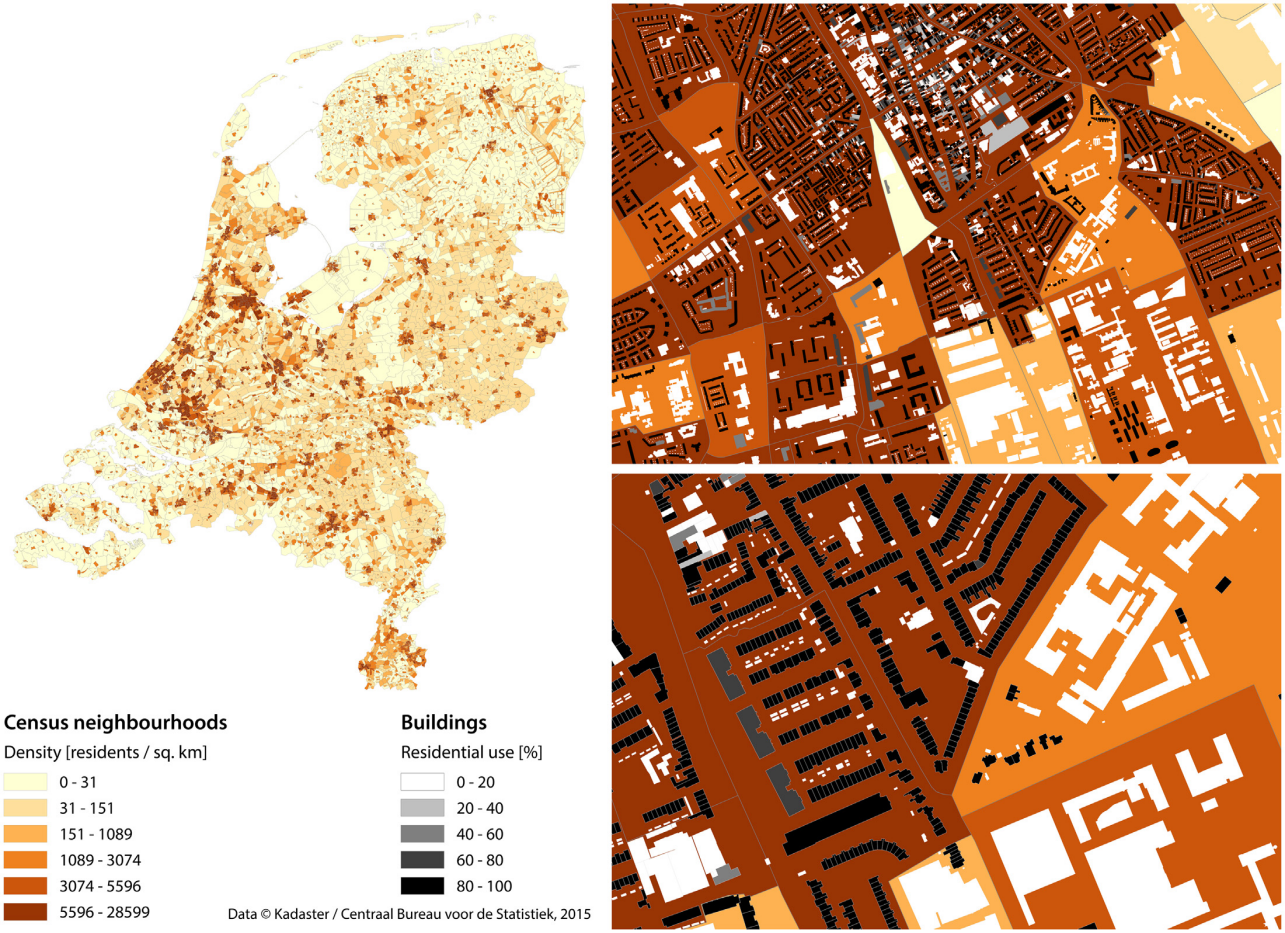
Datasets used in this research: census neighbourhoods with building footprints. (Left side:) The Netherlands divided into more than 12 thousand neighbourhoods; and (right side:) two zoomed-in urban areas, where building footprints are visible along with the information on their use (residential share). Note that the maps on the right side show large variations in population density despite neighbourhoods being similarly urbanised. The less populated areas have many non-residential buildings, e.g. industrial and university buildings, showing that information on their use is crucial, and it significantly impacts the quality of the population estimation. The population density classes are divided into quantiles.

**Fig 2:**
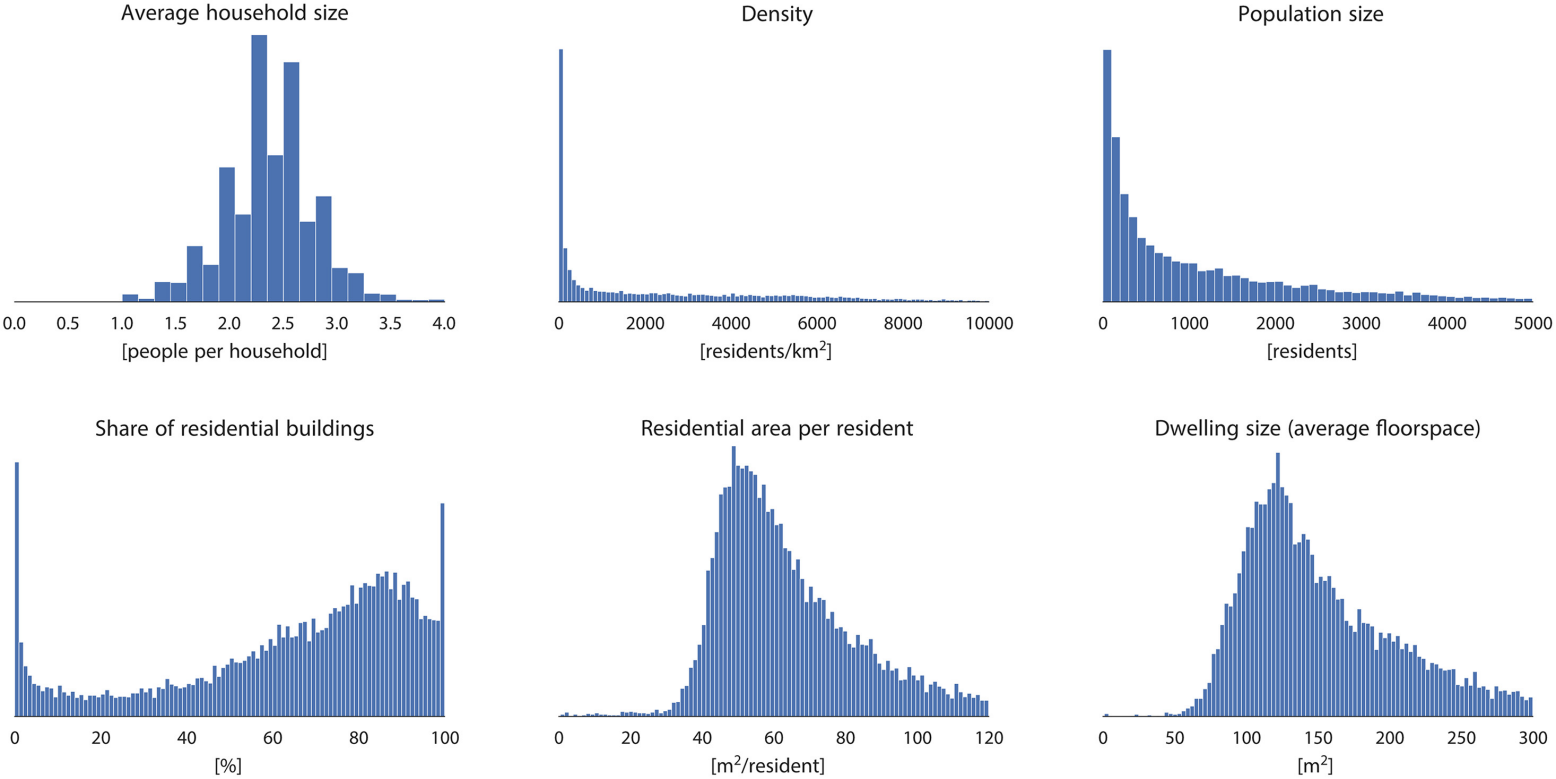
Census neighbourhoods statistics. The plots expose substantial housing differences among the neighbourhoods across the country. Derived from data (c) Kadaster / Centraal Bureau voor de Statistiek, 2015.

The Netherlands is decomposed hierarchically into 12 provinces (not covered further in this paper), 393 municipalities, 2816 districts, and 12237 neighbourhoods. The population of each of the entities is known thanks to the open dataset of Statistics Netherlands—CBS (*Centraal Bureau voor de Statistiek*). As shown in Fig 1, the dataset consists of sets of polygons representing statistical units—the population within each polygon is stored as an attribute. We use this dataset to validate our results, and its subset to train one of the methods. The properties of statistical units across the country vary (see Fig 2), covering widely heterogeneous household sizes, population densities, and dwelling sizes, among others.

### 3D city model of the Netherlands

3D city models are digital representations of the urban environment, focusing on buildings [48–50]. They are used for many different purposes [50], e.g. the prediction of noise pollution [51]. Their key advantage over 2D maps is that they provide volumetric data, which is beneficial for applications that take advantage of the height or volume of buildings, such as energy demand estimations [52, 53] and visibility analyses [54, 55]. Population estimation is clearly such a case, as high-rise residential buildings are very likely to contain more inhabitants per unit area than low-rise buildings.

**Fig 3:**
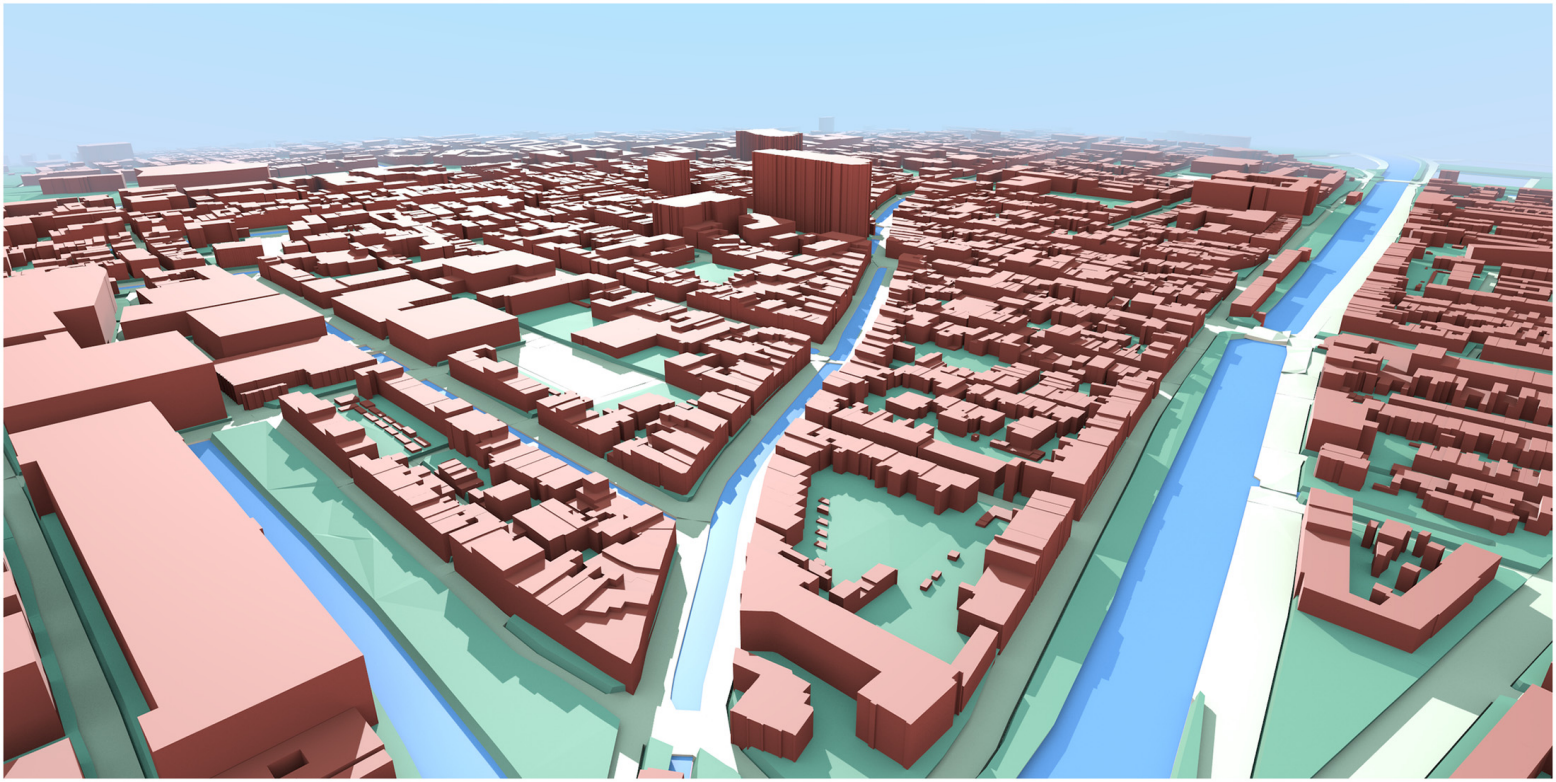
Example of the 3D city model. This example shows a part of the city of Delft, constructed from open data of the Government of the Netherlands ((c) Kadaster and (c) Actueel Hoogtebestand Nederland; see S3 Fig for the illustration of the elevation data).

3D city models can be created with many different techniques, e.g. from airborne laser scanning, and considerable work has been devoted to their automatic generation [56–58]. In this study, we generate a country-wide 3D city model by combining two open datasets from the Netherlands government: (i) building data from the national register of addresses and buildings (BAG—*Basisregistraties Adressen en Gebouwen*, which is collected and maintained by each municipality, and disseminated as country-wide dataset through the national portal of *Kadaster*, the national mapping agency of the Netherlands; and processed by the NLExtract project)—containing the base geometry, building use, and floorspace information (see Fig 1); and (ii) elevation data—the Height Model of the Netherlands (AHN—*Actueel Hoogtebestand Nederland*), which contains 639 billion elevation points covering the whole country (see S3 Fig for an illustration). The 3D model creation is done using a process called extrusion, where the building footprint is lifted to a certain height to obtain a simple volumetric model [59, 60], yielding so-called block models of buildings (LOD1 according to the CityGML standard [61, 62]). For this purpose we have used the software 3dfier, developed by our group and released under an open-source licence (https://github.com/tudelft3d/3dfier). The software analyses all elevation points whose projection is within the footprint of a building, and determines the elevation at the building base and a single value for the height for the building. The height of the building is set to the median of all elevation points, which is considered optimal for building volume estimations [63]. A visual representation of these building block models is given in Fig 3.

**Fig 4:**
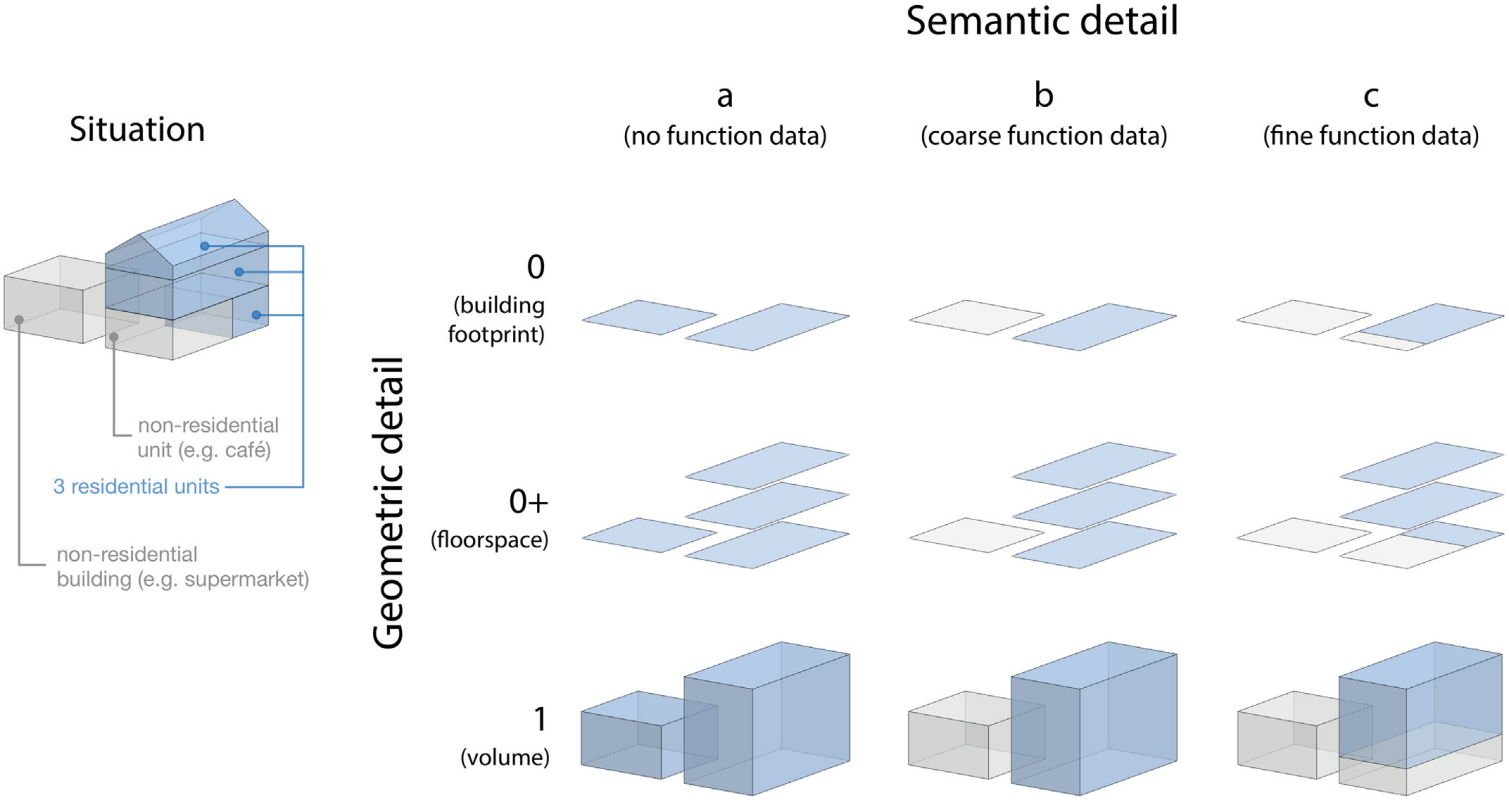
Multi-LOD data used for the experiments. Different granularities, which reflect the different grades of data available in practice. The blue space indicates residential space (proxy for population) as considered for each LOD, which differs depending on the geometry and semantics, and ultimately affects the performance of the methods. In our work we benchmark the performance of each grade of the data for the purpose of estimating the population.

3D city models come in different levels of detail (LODs) and with heterogeneous quality [62, 64, 65], both in terms of geometry and in terms of semantic information (e.g. a building’s use) [66]. Thus, in order to test how different LODs of a 3D city model affect the population estimations, we construct 9 different LODs using various combinations of different levels of detail in a building’s geometric and semantic information (Fig 4). In this way, we can directly compare the quality of the estimations given by the area-based (footprint and floor area) and volume-based approaches.

We consider three geometric LODs: (LOD0) 2D building footprints (the traditional area-based approach without height measurements); (LOD0+) building floorspace (area-based approach in which the vertical extent of the building is available); and (LOD1) volumetric 3D block models (from which the volume of a building can be calculated). For LOD0+, we rely on accurate indoor measurements from the Dutch cadastre, which is a dataset that is rarely available elsewhere. However, it should also be noted there is recent work focused on its automatic reconstruction [67, 68].

The general hypothesis used in this paper, and in related work, is simple: the larger the building, the more people reside in it; and the larger the living capacity of a district, the more populous it is. However, we argue that other building properties should be taken into account as well. The occupancy of a building also depends on its type, e.g. a cathedral, indoor arena, or a factory can be very large but at the same time they house zero inhabitants. Therefore, only residential buildings must be taken into account. This is further complicated by mixed-use buildings, which are composed of non-residential and of residential units, e.g. a three-storey building, where the ground floor is occupied by non-residential space (e.g. a restaurant and a shop), and the remaining two floors by residential units (fairly common in the Netherlands). However, such information is not always available, hence we pay special attention to the semantic aspect of data. Therefore, for the semantic part, we distinguish three levels of detail: (a) no data about the function of the building, and hence all buildings are treated equally; (b) a building is either residential or non-residential [42, 69]; and (c) fractional building use, where the share of the residential use within a building is known.

The possible combinations of the three geometric LODs and the three semantic LODs result in the 9 LODs used in this study, e.g. LOD1^b^ denotes a block model with the singular information on the building use.

### Existing methods for population estimation

**Fig 5:**
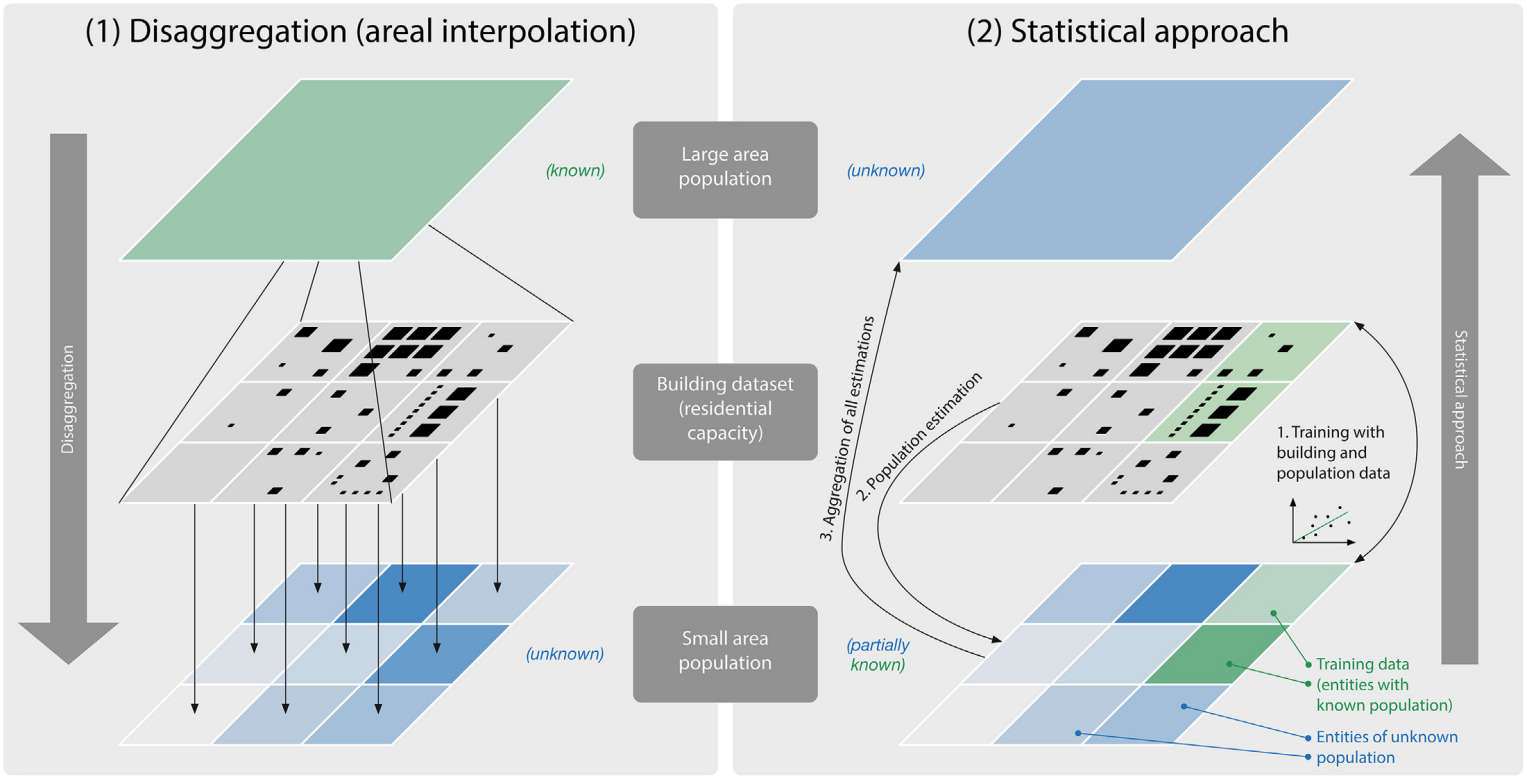
The two population estimation methods used. In this study we employ both methods, and for the residential capacity we use three different indicators in parallel: building footprint area, floorspace area, and building volume. Our work determines the usability of each of the type of geographic information for this purpose.

The estimation of population with GIS data and techniques has been extensively reviewed by numerous authors [22, 41, 70–72]. Generally two groups of methods are recognised [70], both of which are used in this paper (Fig 5):

1. Disaggregation (areal interpolation): this is a top-down approach where the population of a larger administrative unit or zone (e.g. region, municipality, census district) is distributed across smaller units (e.g. neighbourhood), usually by weighting it according to different factors which hint at the population [73–78]. This approach is typically used when the population of a large entity is known (e.g. a city), but the one of its composing entities is not known (e.g. its neighbourhoods).
  The disaggregation can be done by simply distributing the population among administrative sub-zones, but it also can be aided by dasymetric mapping to shape smaller surfaces in such a way that variation within each surface is minimised [79]. This is especially useful when the smaller units are political subdivision of the larger (parent) unit often found in choropleth maps (e.g. interpolation from a province to the containing municipalities), because such regions may contain variations in the population density. Dasymetric mapping therefore results in (sub-)units that are more homogeneous [80–83].
2. Statistical modelling approach: first the relationships between population and socio-economic and morphological variables associated with the population density are inferred, e.g. land use [9, 84], proximity to transportation network [71], and distance from the central business district [85]. The deduced relationships are then applied to estimate the population count of unknown areas. In this approach multiple linear regression is most commonly used. The advantage of this bottom-up approach is that a sampling census has to be carried out for only a small area. It is useful in the scenario when only the population of a subset (e.g. a city) of a large area (e.g. a province) is known.

### Our proposed method using 3D city models

For our population estimation study, we test three indicators to determine the disaggregation weights and the statistical relationships: (i) area of the 2D building footprints (in m^2^), (ii) area of the building floorspace (in m^2^), and (iii) building volume (in m^3^). Each of these is tested at three levels of semantic detail, resulting in the 9 aforementioned LODs of the input datasets.

**Fig 6:**
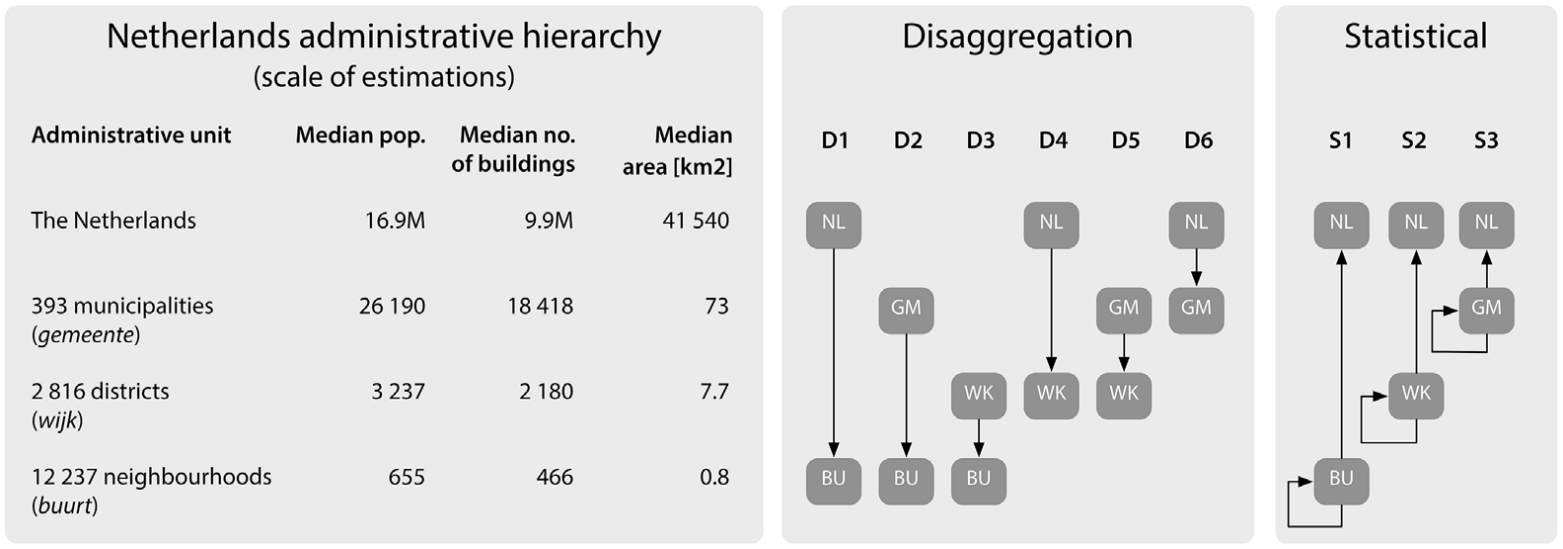
The Dutch statistical hierarchy, and our hybrid multi-scale approach. The hybrid approach refers to both the disaggregation and statistical approach, while multiple scales refer to the level of the statistical units. Statistics of the units obtained from data (c) Kadaster / Centraal Bureau voor de Statistiek, 2015. The provinces are not shown because they have not been considered in our work, and the data refer to the situation in 2015.

In order to diminish residential and socio-economic variations across a large area, but also to test the performance of different estimation scenarios, we use multiple scales of estimations, as shown in Fig 6. In the disaggregation approach 6 scales are analysed:

**D1** Disaggregation from the country level to its 12237 neighbourhoods.
**D2** Disaggregation from each of the 393 municipalities to their 12237 neighbourhoods.
**D3** Disaggregation from each of the 2816 districts to their 12237 neighbourhoods.
**D4** Disaggregation from the country level to its 2816 districts.
**D5** Disaggregation from each of the 393 municipalities to their 2816 districts.
**D6** Disaggregation from the country level to its 393 municipalities.

On the statistical side we use a random subset of 10% of each statistical level to determine with ordinary least squares the relationships between building space and population, and apply them for three different experiments:

**S1** Estimation of the population of the test neighbourhoods (i.e. the remaining 90%).
**S2** Estimation of the population of the test districts (i.e. the remaining 90%).
**S3** Estimation of the population of the test municipalities (i.e. the remaining 90%).

Furthermore, in the statistical approaches (S1, S2, and S3) we also estimate the population of the Netherlands. This means that we test the suitability of carrying out the census for 10% of the country (training dataset), and estimating the population of the rest of a country (test dataset).

In each of the 9 approaches we carry out separate experiments with the data in the 9 different LODs. This results in a total of 108 experiments.

As in related work [44, 86], we ignore very small buildings (footprint smaller than 20 m^2^) such as sheds, garages, etc. which are unlikely to be inhabited (visible in Fig 1 as tiny white footprints in the overly residential areas).

## Results and Discussion

### Performance and observations

We perform the experiments, and compare them to the actual values, as observed in the governmental census dataset (CBS). We use percentage error because we are dealing with different scales of data (e.g. an error of 1000 residents is not of the same magnitude on the neighbourhood or city level). Furthermore, because of large errors in some statistical units (explained later), instead of the usual mean absolute error and root-mean-square error we use the median absolute error. As in related work [71, 87], we observe that estimations in areas with small populations is prone to a high relative error (see S1 Fig), hence medians are a good option here. The results of all experiments are given in Table 1. Because of many different models and types of data, we focus on the most important results only, however, the elaborated observations are similar with the rest of the models. It should be noticed that both the disaggregation and statistical approach exhibit congruent behaviour in most cases.

**Table 1:** Median absolute percentage errors in the population estimates resulting from our experiments.

	^a^	^b^	^c^	^a^	^b^	^c^	^a^	^b^	^c^
	**(1) Disaggregation**								
	**D1 (n = 12237)**			**D2 (n = 12237)**			**D3 (n = 12237)**		
0	61.9	41.9	42.4	53.9	25.5	25.7	42.7	17.7	17.7
0+	39.8	20.8	20.8	37.2	16.2	16.4	29.1	12.0	12.0
1	56.4	25.5	25.8	53.0	20.8	20.7	42.4	15.6	15.3
	**D4 (n = 3237)**			**D5 (n = 3237)**			**D6 (n = 393)**		
0	56.5	37.7	38.2	34.3	15.5	15.5	32.0	25.3	25.5
0+	25.8	16.9	16.5	21.3	9.3	9.2	13.2	11.5	11.4
1	43.5	20.0	20.5	32.0	11.8	11.9	22.1	13.2	13.2
	**(2) Statistical approach (local units)**								
	**S1 (n = 12237)**			**S2 (n = 3237)**			**S3 (n = 393)**		
0	85.4	42.0	42.2	56.9	53.1	52.8	74.0	38.7	38.8
0+	35.4	18.3	18.5	41.5	28.9	28.5	20.6	12.6	12.2
1	66.8	24.3	24.8	49.8	26.1	28.6	28.9	9.5	9.3
	**(2) Statistical approach (country level)**								
	**S1 (n = 1)**			**S2 (n = 1)**			**S3 (n = 1)**		
0	0.6	1.4	1.4	2.7	5.6	5.7	21.5	1.3	1.7
0+	9.3	2.0	2.2	2.7	0.6	0.5	7.9	1.9	1.9
1	4.1	1.2	1.3	3.1	1.9	1.9	11.7	2.0	1.8

The order of errors in each 3×3 matrix is expressed in the same order as the LODs in Fig 4.

**Fig 7:**
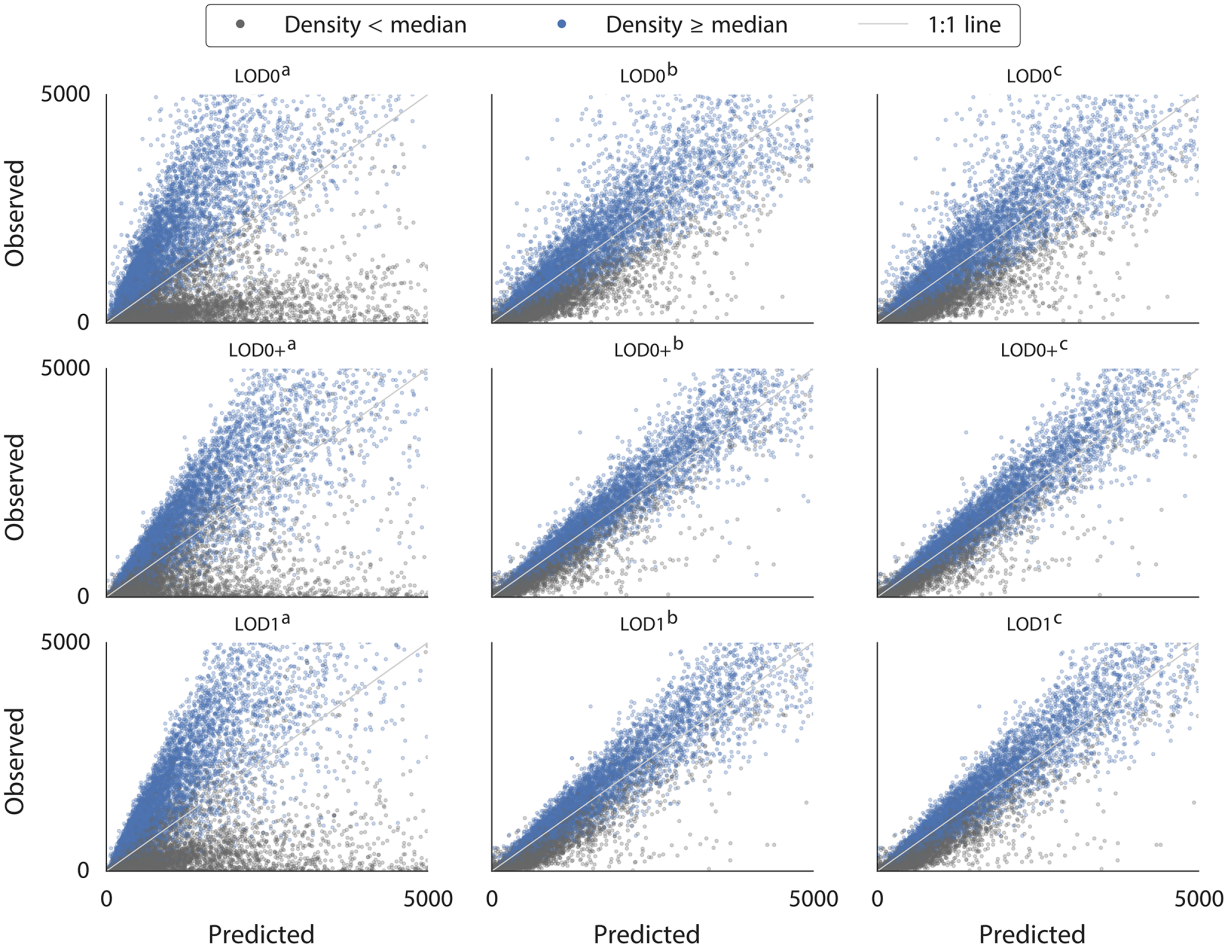
Observed (actual data from the government census) vs predicted scatter plots of the 9 input datasets in the D1 method. The performance of the models depends on the population density of the target area. The lower density refers to areas with the population density lower than the median of all neighbourhoods, and the higher those areas which are denser than the median, indicating urbanised areas. Notice the outliers in the estimations (^a^) that do not take advantage of the semantics—those represent highly industrialised areas without inhabitants or with sparse population. Furthermore, in the experiments carried out with fine-grade data most of the outliers are caused by input data (e.g. mislabelled residential use of a non-residential building) and by districts in which housing standards highly deviate from the average. Observed data (c) Centraal Bureau voor de Statistiek, Den Haag/Heerlen, 2015.

The results exhibit a large degree of variation between the accuracy depending on the approach, level of detail of the data, and the scale of the estimations. The smallest error of the volume-based disaggregation approach is in D5/LOD1^b^ (the disaggregation from municipalities to districts) and it equals 11.8%. The smallest error in the statistical approach was observed in S3 (estimation of the population of cities), resulting in an error of 9.3%. We observe and conclude the following:

- 3D city models and the volume-based approach provide a substantial advantage over traditional 2D maps and the area-based approach because they capture the vertical extent of the building. However, the estimations carried out with 3D models are still less accurate than when using floorspace information. We think that volume does not add value over floorspace because two flats of the same floorspace but of different volumes (e.g. ceiling height of 2.5 m vs 4 m) generally do not host a different number of residents, unlike what the method would predict. It should be noted, however, that floorspace information is difficult to acquire automatically and it is generally not available.
- In most cases, semantic information on the use of buildings provides a substantial improvement in the estimations over data without such information. This helps to exclude non-residential units, which can significantly skew the estimations. Such behaviour is visible as outliers in the scatter plots in Fig 7 (other observations will be discussed in the continuation). Population estimation without information on the building function is practically unusable in most cases, especially in industrial neighbourhoods (in our experiments we have seen overestimations of more than 5000%). In fact, the results show that in this use case, semantic information is typically more important than the geometric detail (e.g. cf. error of 41.9% in D1/LOD0^b^—semantically enriched 2D footprints vs error of 56.4% in D1/LOD1^a^—plain 3D buildings).
- While semantic data is crucial, it appears that there is inconsistent added value of the detailed (fractional) semantic information versus only binary information. It seems that the difference between binary and fractional semantic information becomes negligible at the neighbourhood level. In fact, in some estimations (e.g. D4/LOD1) the estimations with fractional semantic information (D4/LOD1^c^) are slightly less accurate than when using binary semantic information (D4/LOD1^b^).
  In the floorspace data (LOD0+) there is generally a small improvement of using fractional semantic information rather than binary. A possible reason is that the volume-based estimations are more sensitive to errors in the input dataset.
  For the purposes described in this paper, it does not seem worthwhile to collect detailed building usage, as the binary information suffices. Because such information may be automatically derived from the building morphology, aerial imagery, land use maps, etc. [88–94], this insight is beneficial for estimations that need to be carried out on a large extent where cadastral data is not available.
- Different scales of estimations show different performance and different suitability for the different methods. In the disaggregation, the method works best in hierarchically close units: compare D3 (districts to neighbourhoods) with D1 (country to neighbourhoods). This is because such relations exhibit less difference in housing variations. Furthermore, it seems that disaggregating data to units higher in the hierarchy is more accurate than to units of a finer scale, because larger units such as districts and municipalities capture larger residential differences than small neighbourhoods, i.e. the variation among smaller units is greater than that among larger units. For example, two municipalities may have equal population but within municipalities the population differences among districts may be relatively large (e.g. rural vs urban zones). On the other hand differences among neighbourhoods in a district may be small.
- The statistical approach is of comparable accuracy to the disaggregation because it is also based on coefficients uniform for the whole country, which hide massive disparities among different neighbourhoods and provinces, and it is therefore equally affected by the differences in living standards and residential choices.
  However, for the largest extent (country), the statistical approach is impressively accurate: in the S1/LOD1^b^ experiment (statistical approach applied on neighbourhoods with the semantic volume-based LOD1 block model) the population of the Netherlands based on a subset of 10% neighbourhoods has been estimated to 17 100 292, just a 1.2% overestimation from the true figure. The floorspace-based (LOD0+^b^) data fares even better with a deviation of 0.5% in S2. This finding gives confidence in the use of 3D city models for estimating the population of large areas such as countries, especially in developing countries since the data required for such estimations can be derived automatically and remotely from airborne sensors [95]. However, it should be noted that the model S1/LOD0^a^ (building footprints without information on the building use) performed best with an error of 0.6%. It is hard to explain the reason why in this particular model lesser data gave better results, because all errors (induced by different LODs, uncertainty in the input data, different residential choices, etc.) are aggregated in a single number that cannot be decomposed.
- We have noticed that the models tend to overestimate the population in rural areas, and underestimate it in urban areas (see the coloured points in Fig 7). This finding is similar to the observations in related work [38]. The differences are caused by the varying utilisation of living space, which differ between less and more densely populated entities. We use this finding in the succeeding sections for additional insights and we take advantage of it to improve the statistical approach (models S1, S2, and S3).

### Sources of error

After analysing the errors we observe different causes of errors. The residential differences (e.g. residential space per resident) is the principal cause of the residuals (the errors very strongly correlate with the average space per resident; *r* > 0.99). There is a variable level of occupancy and variable utilisation of space within each building, i.e. living space per inhabitant considerably varies based on social, economical, and other factors. Some households live in large houses, while others in small studios and dormitories, rendering significant differences in the residential density [43], and presenting a problem for population estimation with remotely sensed data [46]. Furthermore, these differences are also caused by non-residential space within residential units, such as storage rooms, utility rooms, common rooms, gyms, garages, etc., which increase the building size and considered dwelling space, but due to the shortcomings of the data cannot be accounted as non-residential space. It is usually not possible to assume that these characteristics are equally distributed in each entity, as they are not constant among different neighbourhoods and also on larger extent such as among municipalities [68, 96]. This fact is also visible in Fig 2. Therefore it is important to consider different environments when calibrating the method, and accept imperfections as one model cannot fit all situations within a large area such as a province or country.

We had expected that these differences would cancel out within the statistical entities (since one typically contains hundreds of houses, see Fig 1), however, the difference between units, including larger ones such as cities, is still gross. One would assume that a city contains a fair diversity of different configurations, but it turns out that each city has a unique setting which cannot be applied to another one.

Furthermore, another variation of the dwelling density is caused by vacant residential buildings (e.g. empty houses for sale, vacation homes). In our method we can only assume that the vacancy rate is homogeneous in our area of study, consistently with other researchers (e.g. when estimating the energy demand [97]), however, that assumption might deviate from the reality.

When using the data without information on building use (i.e. LODx^a^) many large errors were found in industrial neighbourhoods with huge building volumes, highlighting the importance of using semantics. When using the semantically enriched buildings, the results improved substantially. However, errors in the input data on building use have also caused errors in the estimation of the population. For instance, we have noted that in an industrial neighbourhood a large factory was mislabelled as a residential building, so the population has been pointlessly disaggregated in an empty building, inducing a substantial error. The input datasets that we used were very accurate [98, 99], but occasional small errors induced gross errors in the estimations. Furthermore, it is worthwhile to mention that there were peculiar cases which also caused discrepancies, such as a small neighbourhood with a prison as its sole building. Its inmates are counted as residents in the CBS dataset, but the prison building is not classified as residential in the cadastral dataset, hence the estimate of the neighbourhood exhibited a large error—the population was predicted to be 0, while in reality it is 75.

The 3D geometric aspect (calculated volume) may induce errors to the estimations as well. It has been suggested [64] that geometric errors in 3D city models (e.g. inconsistencies caused by vegetation in the elevation dataset) may substantially influence spatial analyses, especially the computation of the volume [100].

The related work in analysing error propagation in population estimation is limited to 2D [101–103]. For future work it would be interesting to investigate the influence of errors in the input data when using volume-based approaches.

### Analysis of errors and enhancement of the statistical approach

We have analysed the errors with demographic and other indicators for each statistical unit in order to understand them better and to potentially improve our methods.

We have analysed the income of each neighbourhood and did not find a correlation with errors. We had presumed that low-income neighbourhoods might have less space per resident, as income may be related to living standard and may drive residential purchasing choices. However, that is not the case, because there are cheap but large country-side properties, and expensive small flats in cities such as Amsterdam, invalidating our assumption.

**Fig 8:**
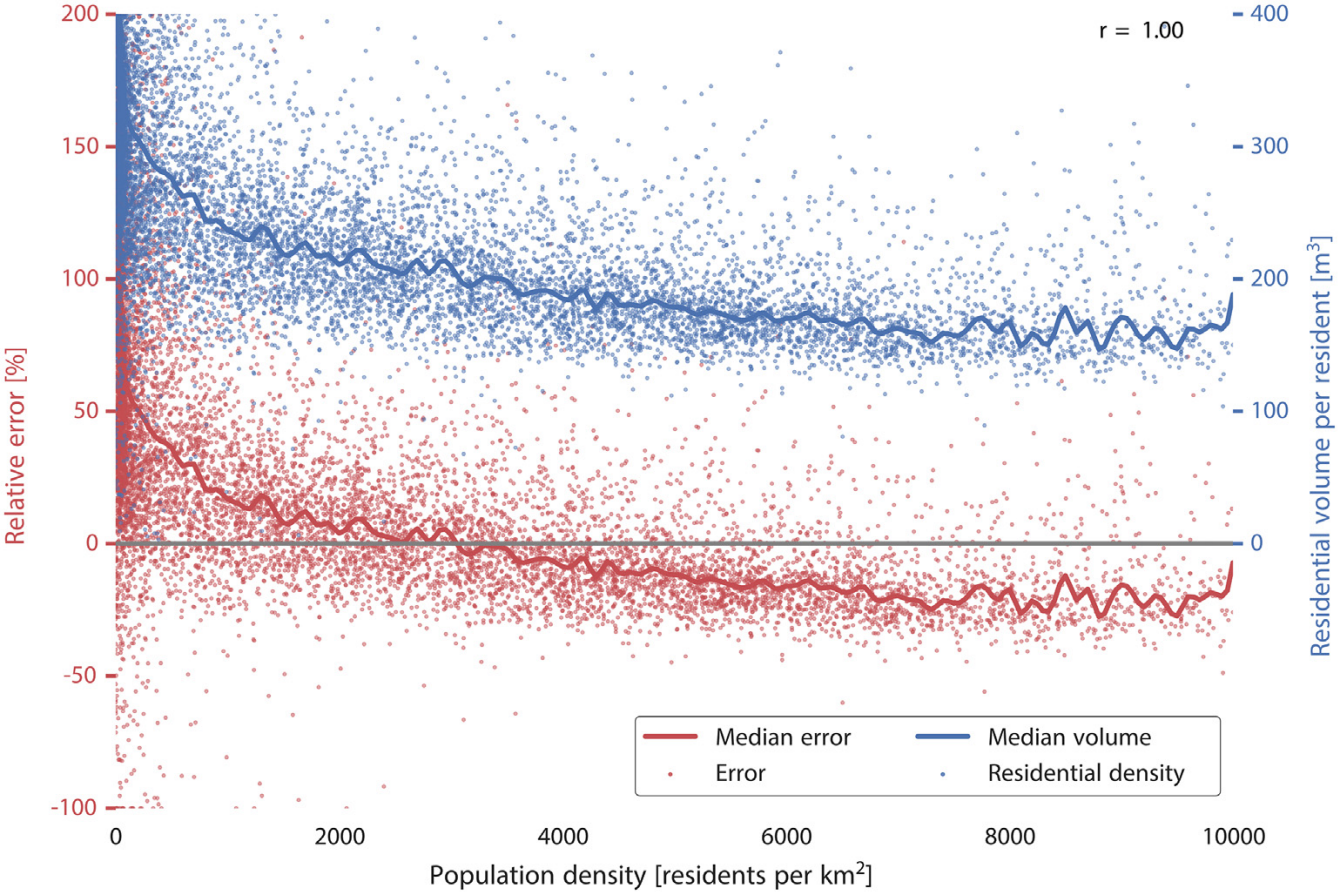
The relations between the errors, population density, and living space per statistical neighbourhood. The errors in the model are from the experiment D1/LOD1^c^. Data (c) Kadaster / Centraal Bureau voor de Statistiek, 2015.

In the previous section we have noted the particular behaviour of errors with respect to the different population densities of estimated areas. There is a clear difference between more and less urbanised areas caused by the different utilisation of dwelling space (see Fig 8). It is clear that the data on the population density could be used to improve the estimations, but as such it is not available prior to the estimation of population (otherwise we would not need to conduct the estimations).

However, we have realised that there is another indicator that it is associated with the population density, and which is available prior to the estimations: the average building height in a neighbourhood is associated with the population density (see S2 Fig), and consequently to the living space. Therefore, for each neighbourhood we have calculated the average building height (easily available since we have 3D city models), and we have incorporated it in our multiple linear regression model (which now contains two variables: the total building space in the statistical unit, and the average height of buildings in the unit). We have not applied this enhancement to the LOD0 approach in which vertical measurements are not available.

The statistical experiments show that there is an improvement to the models: a reduction of errors by a few percent on average has been observed in the models S1, S2, and S3. Note that the results presented in the previous section are of those with the enhanced models, and that the disaggregation method was not enhanced because of its inherently different approach in which there is no training data.

While we believe that the presented prediction models *might* be further augmented to improve the estimations with additional variables and 2D GIS data such as land use, in this paper we have used only 3D models to determine how accurate the predictions can be if relied solely on them. Adding such additional variables is avoided because of a contradictory situation: if such data is available, it is likely that accurate census data is also available, rendering such estimations unnecessary.

### Conclusions and outlook

In this study we have used a 3D city model to estimate the population of 12.2 thousand neighbourhoods, 2816 districts, and 393 municipalities in the Netherlands, and of the Netherlands itself. Our results indicate that in certain circumstances 3D city models can give a good approximation of the population, and that, in most cases, 3D city models add value over traditionally used 2D datasets, but also that they are not accurate enough to replace accurate census techniques employed by governments. Furthermore, there were certain instances when 2D data (even without the information on building use, e.g. S1/LOD0^a^) performed better than 3D data, which is beneficial because such data is simpler to acquire. The main reason why this method is useful is because it does not require expensive and time consuming field surveys and other means of collecting population counts as the data can be acquired automatically and remotely, and it can be carried out more frequently, in contrast to official censuses (usually conducted every decade).

One of the strengths of our work over previous studies is that we carried out a country-wide analysis, in which differences between neighbourhoods are more emphasised. Our study is multi-LOD (both area-based and volume-based approaches have been evaluated, along with multiple grades of semantic information), multi-scale (for assessing the suitability of mapping statistical units of different sizes), and multi-method (both the weighted disaggregation and statistical approaches have been employed).

Remote estimation of population with GIS could be applied in areas where census information is not available or it is not reliable, and serves two purposes: (1) as a potential solution to estimate the population count of large areas where a census is not available, or as an intercensal estimate; and (2) for refining the population on a finer scale (e.g. disaggregation of an accurate census of a city among its neighbourhoods).

Our approach is easily applicable in other countries. Governments have started to publicly release building footprints and other GIS data [89], and where data is available many 3D city models have been generated [104–107]. Alternatively, 3D city models may be generated from volunteered geoinformation [108], ensuring the applicability of our method elsewhere. While in this study for the building use we used datasets from the cadastre, it is worth noting that such data can also be derived manually from aerial images, and automatically from the building morphology and other characteristics, or from volunteered geoinformation [88–94]. Such an approach provides an enhancement over previous research, since in related work coarse datasets have traditionally been used, e.g. Kressler and Steinnocher [5] and Silván-Cárdenas et al. [41] distinguish residential buildings from non-residential ones with a zoning map.

Concerning the first application, estimating the population count of large areas where a census is not available, in the 21st century there are still many places around the world where the census has not been carried out in decades, and such remote sensing methods can help to bridge the gap [109, 110]. For instance, Myanmar did not have a reliable census until two years ago, and in the meantime the authorities were dealing with information which turned out to be significantly erroneous [111, 112], something unthinkable in developed countries nowadays. Obviously, low income countries cannot boast about 3D city models, however, with the development of remote sensing technologies, and surge of volunteered geoinformation and their quality [113, 114], the generation of 3D city models is becoming increasingly simpler and cheaper [107, 108, 115]. Therefore we expect that in the near future country-wide 3D city models will not be a luxury exclusive to developed countries.

With respect to the second purpose, the derived data on the number of residents on a finer scale is beneficial for a multitude of applications [116], such as disaster management (e.g. in flooded areas) [117, 118], analysing accessibility [119], public health [1, 120], crime mapping [121], environmental risk [80, 122], infrastructure planning and transportation sustainability [123], epidemiology [124], territorial classification [125], assessing exposure to noise [51, 126, 127], optimising network coverage (e.g. television) to cover more people [128, 129], for finding areas for landing of stratospheric balloons [129], marketing strategies [44], estimating the quantity of waste [130], estimating energy consumption [131], and in urban simulations [132].

We have also discovered that this method can also be used to detect potential errors in authoritative census and building data (e.g. we have detected erroneous semantic information for some commercial buildings by analysing the large errors in population estimates). Furthermore, we envisage that such method could be used for detecting false residencies (e.g. a large number of people registered in a particular neighbourhood for tax-related reasons, triggering an alert by the population that exceeds the housing capacity in that area).

The results indicate that the estimations are hampered by socio-economic disparities between neighbourhoods, and that population estimation is more reliable when focused on statistical units with a closer proximity. However, this limitation does not seem to affect the estimation of the national population, in which case our method has particularly excelled.

For future work it would be worthwhile to advance the sampling method of the training data in the statistical approach to investigate whether that leads to more accurate estimates. For instance, stratified sampling [133] could be employed instead of the simple random sampling which is used now. Such sampling method could stratify entities based on different characteristics obtainable from 3D city models, such as predominant building types in a neighbourhood, and apply different statistical models to each stratum.

## Supporting Information

S1 Fig Less populated districts exhibit large relative errors, promoting the use of medians. In relative terms, the estimation is more accurate when carried out in more populous areas. These are the results from the experiments S1/LOD1^c^. The two histograms show the data divided in two bins (the left one of the statistical units with the population smaller than the median value of all units (710 residents), and the one on the right the units with the population higher than the median). Not to be confused with Fig 8 which shows the relation of errors to the population density (however, notice that in this case as well the methods tend to underestimate the population in more populated areas).
(TIF)

S2 Fig Association of the population density and vertical extent of the neighbourhood. While the population density is not available for adjusting our models, we have taken advantage of the vertical extent which hints at the population density, and in turn helps in adjusting the prediction between urban and rural areas.
(TIF)

S3 Fig Elevation dataset (AHN) used to generate the 3D city model. The point cloud was obtained with airborne laser scanning, and the colours represent the elevation. The spatial extent and angle of view correspond to the one shown in Fig 3. The accuracy of the points is within a few centimetres [98]. The whole dataset contains 639B points [134]. Data (c) Actueel Hoogtebestand Nederland.
(TIF)
