# Supplementary figures and images for: Population Estimation Using a 3D City Model: A Multi-Scale Country-Wide Study in the Netherlands
Source: PLoS One. 2016 Jun 2;11(6):e0156808. doi: 10.1371/journal.pone.0156808 (PMC4890761; doi:10.1371/journal.pone.0156808)

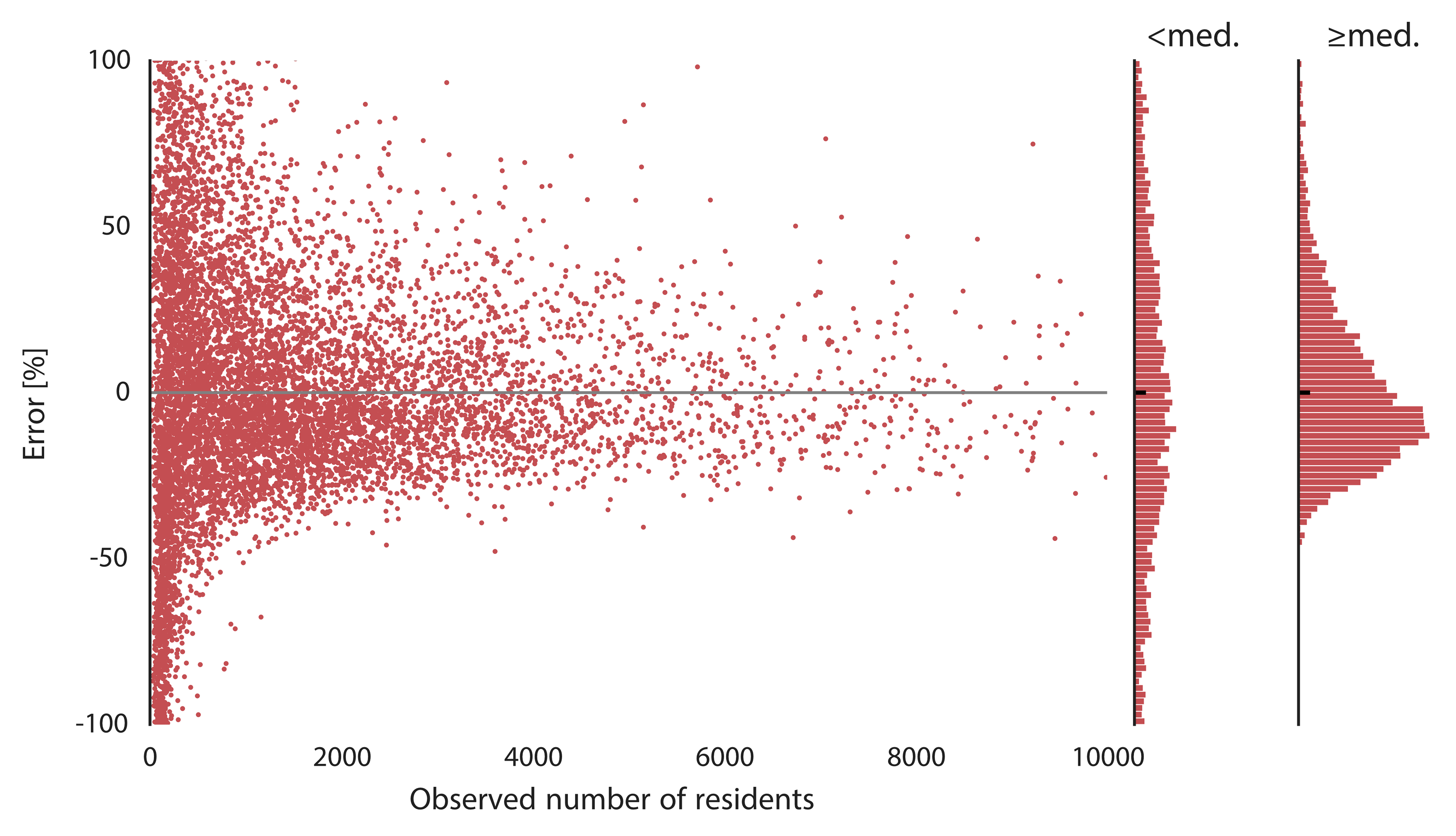

Supplement: S1 Fig — In relative terms, the estimation is more accurate when carried out in more populous areas. These are the results from the experiments S1/LOD1c. The two histograms show the data divided in two bins (the left one of the statistical units with the population smaller than the median value of all units (710 residents), and the one on the right the units with the population higher than the median). Not to be confused with Fig 8 which shows the relation of errors to the population density (however, notice that in this case as well the methods tend to underestimate the population in more populated areas). (TIF) [file pone.0156808.s001.tif]

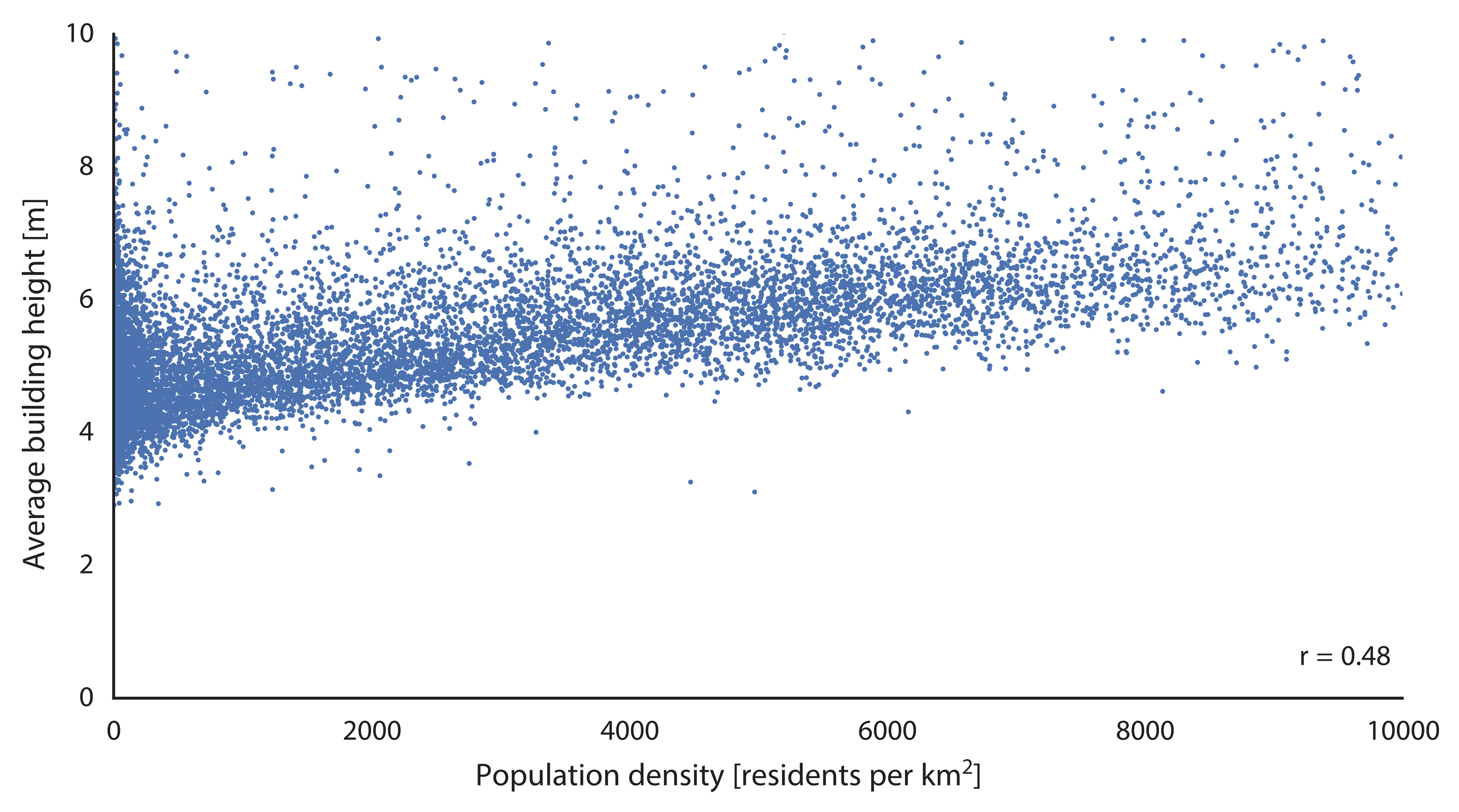

Supplement: S2 Fig — While the population density is not available for adjusting our models, we have taken advantage of the vertical extent which hints at the population density, and in turn helps in adjusting the prediction between urban and rural areas. (TIF) [file pone.0156808.s002.tif]

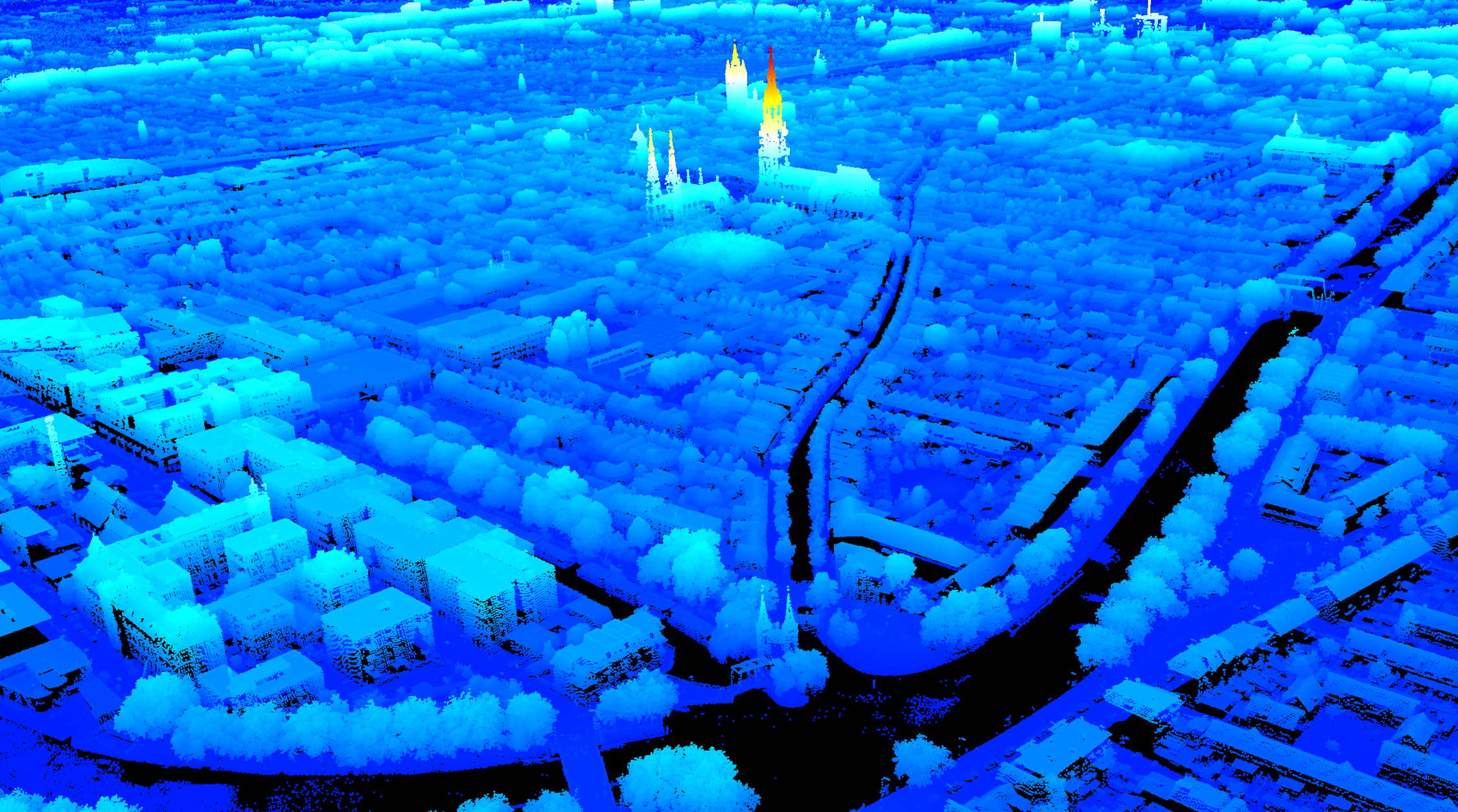

Supplement: S3 Fig — The point cloud was obtained with airborne laser scanning, and the colours represent the elevation. The spatial extent and angle of view correspond to the one shown in Fig 3. The accuracy of the points is within a few centimetres [98]. The whole dataset contains 639B points [134]. Data (c) Actueel Hoogtebestand Nederland. (TIF) [file pone.0156808.s003.tif]
